# The complementary role of PSMA expression and [^18^F]FDG PET/CT in predicting thyroid cancer outcome: from black and white to shades of gray, in the era of precision oncology

**DOI:** 10.1186/s13550-023-01004-2

**Published:** 2023-06-01

**Authors:** Martina Sollini, Margarita Kirienko, Luca di Tommaso, Cristiano Pini, Fabrizia Gelardi, Salvatore Ariano, Andrea Gerardo Lania, Gherardo Mazziotti, Giuseppe Mercante, Arturo Chiti

**Affiliations:** 1 Department of Biomedical Sciences, Humanitas University, Via Rita Levi Montalcini 4, 20072 Pieve Emanuele, Milan Italy; 2 Nuclear Medicine, IRCCS Humanitas Research Hospital, Via Manzoni 56, 20089 Rozzano, Milan Italy; 3 Fondazione IRCCS Istituto Nazionale dei Tumori, Milan, Italy; 4 Pathology, IRCCS Humanitas Research Hospital, Rozzano, Milan Italy; 5 Endocrinology, IRCCS Humanitas Research Hospital, Rozzano, Milan Italy; 6 Otorhinolaryngology, IRCCS Humanitas Research Hospital, Rozzano, Milan Italy; 7 Present Address: School of Medicine and Surgery, University of Milano-Bicocca, Monza, Italy; 8 Present Address: Faculty of Medicine and Surgery, Vita-Salute San Raffaele University, Milan, Italy; 9 Present Address: Department of Nuclear Medicine, IRCCS Ospedale San Raffaele, Milan, Italy

**Keywords:** Thyroid cancer, [^18^F]FDG PET/CT, Prostate specific membrane antigen, Radioiodine

## Abstract

**Background:**

The value of Prostate Specific Membrane Antigen (PSMA) in thyroid carcinoma (TC) is still unknown. We aimed to test the potential complementary role of PSMA expression and 2-[^18^F]fluoro-2-deoxy-D-glucose ([^18^F]FDG) uptake on PET/CT as biomarkers for TC outcome prediction.

**Materials and methods:**

From a retrospective cohort of TC patients we selected those fulfilling the following inclusion/exclusion criteria: thyroidectomy in our Institution, available primary tumor tissue PSMA immunostaining, [^18^F]FDG PET/CT and follow-up data. PSMA staining was visually assessed. PET/CT was considered positive in case of [^18^F]FDG uptake higher than the background at the site of TC confirmed by cyto-/histology, and/or follow-up. Disease recurrence, radioiodine refractoriness (RAI-R) and status at last follow-up (LFU) were used as outcome endpoints.

**Results:**

We included 23 subjects. Disease recurrence occurred in 18 patients (median time 11 months, range 1–40); among these 12/18 developed RAI-R (median time 28 months, range 2–221), and 13/18 had evidence of disease at LFU. PSMA expression was negative in 6/23 cases. PET/CT was negative in 11/23 patients (7/11 experienced recurrence). PET/CT was positive in 9/12 patients showing RAI-R and 10/13 cases with evidence of disease at LFU. All patients with positive PET/CT had a positive PSMA immunostaining. Six out of 11 patients with negative PET/CT were positive at immunostaining, showing lower PSMA expression (median score of 30%, range 0–80%) than patients with positive PET/CT. The TC samples without PSMA expression belonged to patients who resulted negative also at PET/CT (3 experienced recurrence, 2 were RAI-R, and 1 had disease at LFU). Four out of 11 patients who resulted negative at PET/CT exhibited very high PSMA expression (≥ 70%) and although 3 of them experienced recurrence, none resulted RAI-R, and only 1 had persistent disease at LFU.

**Conclusions:**

Primary tumor PSMA expression and [^18^F]FDG uptake seem to play a complementary prognostic role in TC. The majority of patients who expressed PSMA recurred. In the intermediate ATA risk class, patients with negative PSMA immunostaining recurred less than patients expressing PSMA. Additionally, although patients with a negative [^18^F]FDG PET/CT had a favourable long-term outcome, PSMA assessment might be useful to timely identify subjects at higher risk of recurrence.

## Background

2-[18F]fluoro-2-deoxy-Dglucose ([^18^F]FDG) is the most commonly used PET tracer. Among radiopharmaceuticals developed to fill the gaps of [^18^F]FDG, those targeting prostate specific membrane antigen (PSMA) resulted accurate in diagnosis and promising in prognostication. The complementary predictive and prognostic role of [^18^F]FDG and PSMA, has been reported in different malignancies [1, 2]. In thyroid cancer (TC), they have been explored [3, 4], but their possible complementary role has not been established yet. Complementary findings detected by PSMA and [^18^F]FDG PET/CT have been reported in small series of radioiodine refractory (RAI-R) TC patients [5, 6]. Ciappuccini et al. [7] found a certain degree of concordance between PSMA and [^18^F]FDG in a cohort of 44 recurrent TC. Moreover, patients with [^18^F]FDG-avid lesions showed higher PSMA expression than those with [^18^F]FDG negative lesions, although no significant differences were observed in terms of progression-free survival (PFS). Despite promising, the evidence on the topic is still limited. Therefore, we aimed to test the potential complementary role of PSMA expression and [^18^F]FDG uptake on PET/CT as biomarkers for TC outcome prediction.

## Materials and methods

From a retrospective cohort of TC patients we selected 23 patients fulfilling the following inclusion/exclusion criteria: thyroidectomy in our Institution, available primary tumor tissue PSMA immunostaining, [^18^F]FDG PET/CT and follow-up data. Demographics, histology, risk of structural disease recurrence assessed according to the American Thyroid Association (ATA) Guidelines [4], and follow-up data were collected (Table 1). Methods for PSMA expression assessment have been previously detailed [8]. Briefly, an experienced pathologist firstly visually dichotomized the cases as negative (≤ 5%) versus positive, further scoring the latter according to the percentage of expression on the whole slide. Re-staging [^18^F]FDG PET/CT, acquired according to EANM guidelines [9] on a Siemens Biograph LS6 scanner (Siemens, Munich, Germany) or a GE Discovery PET/CT 690 (General Electric Healthcare, Waukesha, WI, USA), was considered positive in case of [^18^F]FDG uptake higher than background, at the site of recurrence. Outcome endpoints, calculated as the time between radioiodine treatment and event (or censored datum), were disease recurrence (i.e. macroscopic evidence of disease after thyroidectomy and radioiodine), RAI-R defined according to the ATA Guidelines [4], and status at last follow-up (LFU) which considered the presence (or not) of macroscopic disease at last visit. Frequency tables and descriptive statistics were used to report and analyze data. The study was approved by the Ethics Committee of the IRCCS Humanitas Research Hospital.

**Table 1:** Baseline patients characteristics

	Population (n = 23)
Age, years	
Mean ± standard deviation	56.4 ± 18.6
Median, range	60, 20–80
Sex	
Male	8
Female	15
Histology	
Papillary	16
Poorly differentiated	4
Follicular	2
Papillary + follicular	1
Pathological nodal involvement at diagnosis	
Yes	9
No	7
Unknown	7
Distant metastases at diagnosis	
Yes	9
No	14
Risk of structural disease recurrence according to ATA Guidelines [4]	
Low	2
Intermediate	6
High	11
Not assessable*	4

*All patients with poorly differentiated thyroid cancer

## Results

### PSMA expression

PSMA staining was positive in 17/23 cases (score of 30%, 40%, 60%, 70%, and 80% in 2, 6, 1, 3, and 5 patients, respectively). Table 2 details PSMA expression according to the ATA risk of recurrence.

**Table 2:** PSMA expression according to the ATA risk of structural disease recurrence (n = 23)

ATA risk of structural disease recurrence	PSMA expression	
	Negative (n = 6)	Positive (n = 17)
Low risk	0	2
Intermediate risk	4	2
High risk	2	9
Not assessable (poorly differentiated)	0	4

### [^18^F]FDG PET/CT

**Fig. 1:**
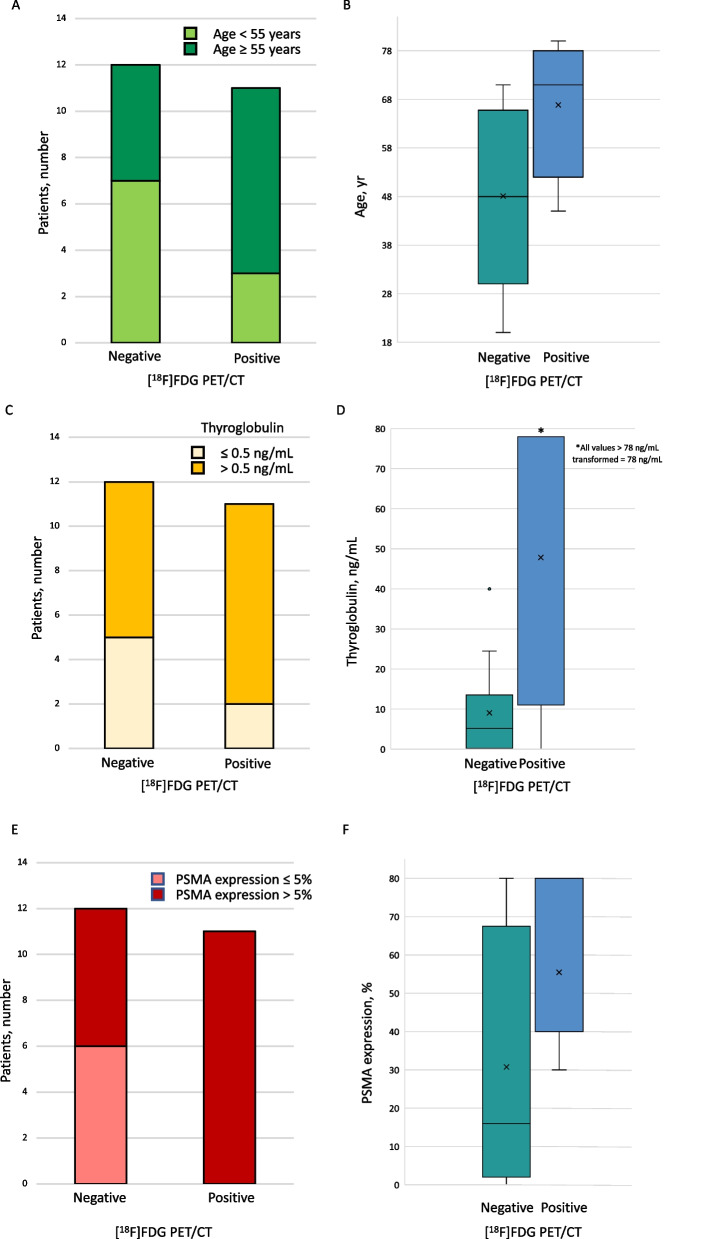
Age distribution (**a**, **b**), thyroglobulin level at the time of imaging (**c**, **d**), and PSMA expression level (**e**, **f**) in the population according to [^18^F]FDG PET/CT results

Re-staging [^18^F]FDG PET/CT was performed because of increased thyroglobulin levels (15/23), as part of follow-up (5/23) or to investigate other conditions (3/23). [^18^F]FDG PET/CT was positive in 11/23 cases. We did not observe differences in terms of gender between [^18^F]FDG positive and negative patients (4 males in each group). Patients with negative scan were younger than those with a positive one (median age 48 years, range 20–71 *versus* 71 years, range 45–80; Fig. 1a, b). Thyroglobulin at the time of imaging differed in patients with negative and positive scan (median 5.2 ng/mL, range 0.03–40 versus 1932 ng/mL, range 0.2–46,000, Fig. 1c, d).

### Comparison between PSMA expression and [^18^F]FDG PET/CT

**Fig. 2:**
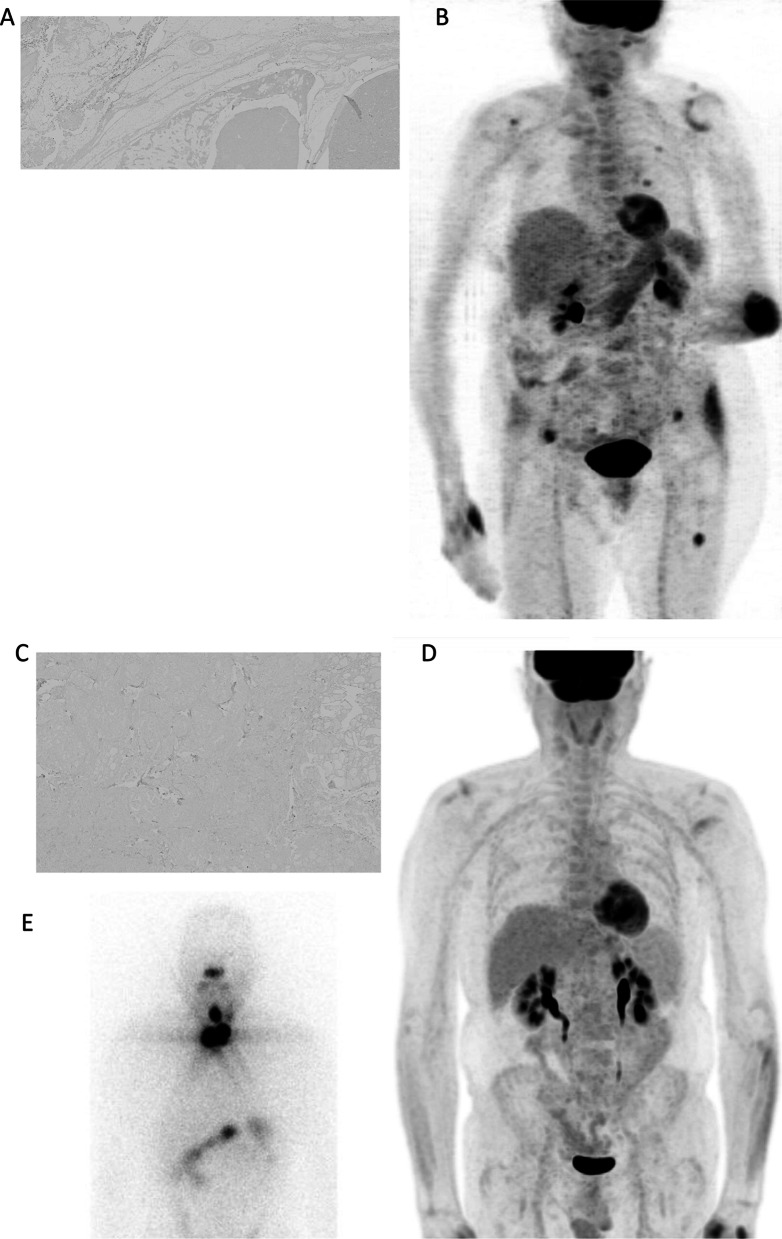
Clinical examples of immunostaining and [^18^F]FDG PET/CT in two patients. In **a**, **b** images of a 71 year-old female patient affected by mixed papillary and follicular thyroid cancer (pT3pN0pM1, margins focally involved). She was classified at high risk of recurrence according to ATA Guidelines. PSMA immunostaining was scored as positive (a, 40% expression) and she had multiple [^18^F]FDG-avid bone and lung metastases (**b**). Disease recurrence occurred six months after treatment. At 16 months patient developed RAI-R, and she had persistent lung and bone disease at last follow-up visit at 38 months (thyroglobulin at last follow-up was 2043 ng/mL). In **c**–**d**–**e** images of a 60 year-old male patient with left papillary thyroid cancer (pT1pN1, margins involved). He was classified at high risk of structural disease recurrence according to ATA Guidelines. He had negative PSMA immunostaining (**c**). Thyroglobulin at the time of PET/CT was 40 ng/mL and even if imaging (**d**) did not show any abnormal [^18^F]FDG uptake, neck disease recurrence was confirmed few days after by Iodine-131 whole-body scan (**e**). At 23 months the patient developed RAI-R, and he had persistent nodal disease at last follow-up visit at 92 months (thyroglobulin of 5.2 ng/mL at last follow-up)

All patients with positive [^18^F]FDG PET/CT had a positive PSMA immunostaining (median score of 40%, range 30–80%; Fig. 2a, b). Half patients with negative [^18^F]FDG PET/CT were positive at immunostaining (Fig. 1e). Patients with negative [^18^F]FDG PET/CT exhibited lower PSMA expression (median score of 16%, range 0–80%) than patients with positive scan (Fig. 1f).

### Comparison between PSMA expression, [^18^F]FDG PET/CT, ATA risk and outcome

Disease recurrence occurred in 18/23 patients (median time 11 months, range 1–40); among these 12/18 developed RAI-R (median time 28 months, range 2–221), and 13/18 cases had disease at LFU (median time 84 months, range 6–221). [^18^F]FDG PET/CT resulted negative in 7/18 patients who experienced disease recurrence (Table 3). Only 1/11 patients classified at high risk did not experience recurrence. Two patients were classified as low risk; both recurred, expressed PSMA and presented [^18^F]FDG-avid disease. Patients with intermediate risk and negative PSMA recurred less than patients with the same risk and positive immunostaining (1 vs. 3, respectively). [^18^F]FDG-avid disease was detected in 9/12 RAI-R and 10/13 cases with evidence of disease at LFU, respectively (Table 4). Patients with positive [^18^F]FDG had a shorter PFS than those with a negative scan who experienced recurrence (6 vs. 11 months). Similarly, disease-free survival (DFS) was shorter in case of positive scan (54 vs. 92 months).

**Table 3:** Disease recurrence according to the risk assessed according to ATA Guidelines [4], [^18^F]FDG PET/CT findings and PSMA expression

Disease recurrence	Risk of structural disease recurrence according to ATA Guidelines [4]	[^18^F]FDG PET/CT (n = 23)		
		Negative (n = 12)		Positive (n = 11)
		PSMA negative (n = 6)	PSMA positive (n = 6)	PSMA positive (n = 11)
Yes (n = 18)	Low	n = 0	n = 0	n = 2
	Intermediate	n = 1	n = 1	n = 0
	High	n = 2	n = 2	n = 6
	Not assessable	n = 0	n = 1	n = 3
No (n = 5)	Low	n = 0	n = 0	n = 0
	Intermediate	n = 3	n = 1	n = 0
	High	n = 0	n = 1	n = 0
	Not assessable	n = 0	n = 0	n = 0

**Table 4:** Outcome results according to [^18^F]FDG PET/CT findings and PSMA expression

Outcome	[^18^F]FDG PET/CT (n = 23)		
	Negative (n = 12)		Positive (n = 11)
	PSMA negative (n = 6)	PSMA positive (n = 6)	PSMA positive (n = 11)
Radioiodine refractoriness			
Yes (n = 12)	n = 2	n = 1	n = 9
No (n = 11)	n = 4	n = 5	n = 2
Evidence of disease at last follow-up			
Yes (n = 13)	n = 1	n = 2	n = 10
No (n = 10)	n = 5	n = 4	n = 1

TC samples that did not express PSMA belonged to patients also negative at [^18^F]FDG PET/CT (Fig. 2c, d): three experienced recurrence (Fig. 2e), two were RAI-R, and one had disease at LFU.

Only 1/3 patients with negative [^18^F]FDG PET/CT who became RAI-R expressed PSMA (i.e. 30%). Three out of 12 patients who resulted negative at [^18^F]FDG PET/CT exhibited very high PSMA expression (≥ 70%): among these two experienced recurrence and one had disease at LFU. The remaining patients with negative scan had low-moderate PSMA expression: two of them (30% and 60% PSMA expression, respectively) recurred and presented disease at last follow-up. The remaining two patients (2% and 40% PSMA expression, respectively) did not experience any event.

## Discussion

Our findings suggest that PSMA expression and [^18^F]FDG PET/CT findings play a complementary role in TC risk stratification. The majority of patients who expressed PSMA recurred (15/18). [^18^F]FDG positive scan was related to high recurrence rate (11/18), risk of RAI-R development (9/12), and presence of disease at LFU (10/13). Moreover, patients with positive scan exhibited higher PSMA expression than patients with negative [^18^F]FDG PET/CT (Fig. 1e, f).. The expression of PSMA, a marker of neovasculature formation [10, 11], has been reported to be related to tumor recurrence in TC [8, 12]; while high [^18^F]FDG uptake, a marker of less differentiated thyroid tumors [13], indicates poorer prognosis [4, 14]. Ciappuccini et al. [7] found a significantly different immunoreactive score in patients with positive PET/CT compared to those with negative scan, and a lower disease-free survival in patients who presented [^18^F]FDG uptake. Collectively, our cohort included a higher proportion of patients who expressed PSMA than those reported by Ciappuccini et al. [7] (17/23 vs. 30/44), especially in case of negative scan (6/12 versus 3/30). Nonetheless, as previously shown [7], we confirmed that a higher number of recurrent patients with positive [^18^F]FDG PET/CT expressed PSMA at a higher level (79% vs. 25%) than those with a negative scan (11/18 versus 4/18). Preliminary data showed that PSMA-targeting imaging might add information to [^18^F]FDG PET/CT, potentially impacting on patient management [6, 15], although detection rates ranging from 25 to 100%, were overall inferior to [^18^F]FDG PET/CT, when compared [16]. We found an absolute agreement between [^18^F]FDG positivity and PSMA expression, while we can speculate a little more in patients with negative [^18^F]FDG PET/CT, with a potential impact of PSMA positivity on disease recurrence rate. However, the numbers are small to draw conclusions and taking into consideration potential selection bias of this retrospective study. Indeed, [^18^F]FDG PET/CT is generally performed in clinical practice in patients with suspected recurrence, as also suggested by thyroglobulin levels in our cohort. Therefore, a high prevalence of recurrence was expected in both patients with positive and negative scan. However, when considering only patients with negative scan, the probability of recurrence in case of PSMA expression was double than in patients with both negative immunostaining and [^18^F]FDG PET/CT (4 vs. 2 and 3 vs. 3, respectively).

Our results suggest that PSMA expression might be an alternative to [^18^F]FDG PET/CT as prognostic biomarker. Moreover, PSMA expression assessment has the advantage of being not expensive, and available at diagnosis with a minimal additional effort from pathologists. Therefore, differently from [^18^F]FDG that—even if recognized as prognostic TC biomarker—is not routinary used probably because of costs and limited direct impact on patient management, PSMA expression assessment can be easily implemented in clinical practice. This would allow to set up at baseline a tailored treatment and follow-up according to PSMA staining findings. Moreover, radioligand therapy might become a valuable therapeutic option to offer to recurrent patients, upon in vivo target confirmation by PET/CT. Notably, in our cohort both patients classified as low risk of recurrence according to ATA Guidelines [4] expressed PSMA (40% and 70%, respectively) and recurred. Additionally, patients with intermediate risk and negative PSMA immunostaining recurred less than patients belonging to the same class risk and PSMA expression (1 versus 3, respectively). If PSMA positivity will be confirmed in dedicated prospective trials as an outcome predictor, immunohistochemistry for PSMA assessment could be implemented in the clinics alongside other pathological data. We have to acknowledge some limitations. Firstly, the retrospective design and the criteria used for patients’ selection possibly affected our results. For some patients, a limited follow-up time was available.

## Conclusions

Primary tumor PSMA expression and [^18^F]FDG seem to play a complementary role in TC. The majority of patients who expressed PSMA recurred. Patients with intermediate risk and negative PSMA immunostaining recurred less than patients who belong to the same class risk and expressed PSMA. Additionally, although patients with a negative [^18^F]FDG PET/CT had a favourable long-term outcome, PSMA assessment might be useful to timely identify subjects at higher risk of recurrence. In the era of precision oncology and personalized medicine, PSMA may represent a powerful tool at our disposal to single out those [^18^F]FDG-negative patients that will nonetheless develop TC recurrence, potentially improving the outcome of this hard-to-manage disease.

## Data Availability

The manuscript represents valid work, and neither this manuscript nor one with substantially similar content under the same authorship has been published or is being considered for publication elsewhere. Martina Sollini had full access to all the data in the study and takes responsibility for the data integrity and the accuracy of the data analysis. Raw data are available on specific request to the corresponding author (https://doi.org/10.5281/zenodo.7808016).

## Abbreviations

[^18^F]FDG: 2-[^18^F]fluoro-2-deoxy-D-glucose
ATA: American Thyroid Association
DFS: Disease-free survival
LFU: Last follow-up
PET/CT: Positron emission tomography/computed tomography
PSMA: Prostate specific membrane antigen
RAI-R: Radioiodine resistant
RAI: Radioiodine
TC: Thyroid cancer
WBS: Whole body scan
